# Effect of modified atmosphere packaging and vacuum packaging on quality characteristics of lamb meat

**DOI:** 10.5194/aab-64-437-2021

**Published:** 2021-10-15

**Authors:** Katarzyna Ząbek, Jan Miciński, Stanisław Milewski, Alicja Sobczak

**Affiliations:** Department of Sheep and Goat Breeding, Faculty of Animal Bioengineering, University of Warmia and Mazury in Olsztyn, Oczapowskiego 2, 10-957 Olsztyn, Poland

## Abstract

The aim of the study was to evaluate the effect of vacuum packaging and modified atmosphere packaging (80 % N${}_{2}$ + 20 % CO${}_{2}$) on the microbial and physicochemical parameters of lamb meat and the sensory properties of cooked meat. Musculus longissimus thoracis et lumborum samples were examined at 10 d intervals (0, 10, 20 and 30 d) during storage at 4 ${}^{\circ }$C. There was no significant effect of the packaging method and storage time used on cooking loss, natural drip loss, lightness, yellowness, and intensity of taste and aroma. An interaction between storage time, packaging method, and mesophilic aerobic bacteria and coliform counts was observed. Storage time significantly affected the number of aerobic psychrotrophic bacteria, redness, pH (P≤0.001), shear force value (P=0.006), and the desirability of aroma (P<0.026) and taste (P<0.01). During the storage time, an increase in red saturation from 11.92 to 13.33 and pH value from 5.69 to 5.80 was recorded. Moreover, the storage method affected sensory properties. Vacuum-packed meat was characterized by higher scores in juiciness, tenderness and taste desirability in comparison to MAP. The obtained results suggest that both packaging methods allow for maintaining high-quality lamb meat during a long period of storage under refrigeration conditions.

## 1 Introduction

Food packaging has multiple functions, such as protection, promotion, identification, convenience and increasing the shelf life of a product (Gómez and Lorenzo, 2012). Vacuum packaging (VP) and modified atmosphere packaging (MAP) are packaging technologies used for fresh meat and processed meat products in order to extend the shelf life (Hur et al., 2013).

The VP extends the storage of chilled meats by maintaining an oxygen-deficient environment inside the pack and in this way it prevents the growth of aerobic bacteria and leads to the slow growth of CO${}_{2}$-tolerant bacteria. However, use of VP leads to changes in the surface color of fresh meat from bright red to purplish red due to deoxymyoglobin formation (Kim et al., 2012; Reis et al., 2016). Mancini and Hunt (2005) state that color is one of the most important features determining consumer acceptability of fresh meat.

As McMillin (2008) notes, MAP is the removal and replacement of the atmosphere surrounding the product before sealing in vapor-barrier materials. It is used to extend the shelf life of meat, maintain its sensory quality and avoid changes in product appearance (Bórnez et al., 2010; Cayuela et al., 2004). Carbon dioxide (CO${}_{2}$), nitrogen (N${}_{2}$) and oxygen (O${}_{2}$) in different concentrations are the most commonly used gases in meat packaging technology (Morales-delaNuez et al., 2009). Each of these gases play a specific role in the conservation process and affect the meat quality (Carrizosa et al., 2017). High levels of CO${}_{2}$ decrease respiration and thus decrease the growth rate of microorganisms (Simpson et al., 2009). According to Simpson et al. (2009), CO${}_{2}$ has greater inhibitory effect at lower temperatures due to the increased solubility of CO${}_{2}$. Some authors noticed that despite a concentration of CO${}_{2}$ close to 100 % extending the storage period due to controlling spoilage bacteria, CO${}_{2}$ also has a negative effect on the appearance, color stability and the texture of raw meat compared with other gas mixtures (Fernandes et al., 2014; Viana et al., 2005). Nitrogen gas has no influence on the packed product and is used as a complement to O${}_{2}$ and CO${}_{2}$ in gas mixtures (Cayuela et al., 2004). The oxygen in gas mixtures is responsible for the bright red color of meat (Fernandes et al., 2014). However, as many authors state, high oxygen concentrations can reduce the shelf life of meat due to color loss caused by the development of lipid and protein oxidation (Linares et al., 2007; Martínez et al., 2005; Ripoll et al., 2013). According to Jeremiah (2001), the optimum color stability of red meat is possible via the use of gas mixtures containing a higher concentration of oxygen in combination with low carbon dioxide content. However Vergara and Gallego (2001) reported that lamb meat packed using gas mixture with the proportions of 40 % CO${}_{2}$ and 60 % N${}_{2}$ maintain suitable color and odor.

Deterioration of meat, and hence changes in it quality characteristics, depends on external and internal factors such as pH, morphological structure of the surface, oxygen availability, contamination of meat with putrefactive bacteria at various stages of the production process, temperature, and the presence and development of other bacteria (Carrizosa et al., 2017; Ercolini et al., 2009; Osés et al., 2013).

The aim of this study was to determine the effect of modified atmosphere packaging (80 % N${}_{2}$ and 20 % CO${}_{2}$) and vacuum packaging methods on the quality characteristics and shelf life of lamb meat during storage.

The research hypothesis assumes that both vacuum and modified atmosphere packaging extend the storage time without damaging the quality of lamb meat.

## 2 Material and methods

The investigation was performed on 12 single-born sucking Kamieniec lambs born at the same time. The animals were fed standard diets recommended by INRA (1988). Until 10 d of age, the lambs were fed exclusively on their mothers' milk. Starting from day 11, they also received meadow hay and CJ^®^ mixture, followed by maize silage offered from day 30. Components of the mixture were as follows: ground barley (40 %), ground wheat (37.5 %), ground maize (10 %), soybean meal (10 %), mineral premix (2 %), fodder chalk (0.2 %), dicalcium phosphate (0.2 %) and salt fodder (0.10 %). Permission from the Local Ethics Committee for Animal Experiments in Olsztyn (no. 31/2009) was obtained for the tests.

### 2.1 Sample preparation

Lambs were slaughtered at the age of 100 d. The animals were slaughtered in a commercial slaughterhouse by mechanical method using a stun gun (Blitz-Kerner) according EFSA (2004). Samples for meat quality assessment were collected from longissimus thoracis et lumborum (LTL) muscle after 24 h of carcass chilling at 4 ${}^{\circ }$C. A factorial design was used: two batches (vacuum and modified atmosphere) × two meat samples in each sample point × four sample points (0, 10, 20 and 30 d of storage). Two methods of meat storage were used: vacuum storage (VP) and storage in modified atmosphere (MAP) gases (80 % N${}_{2}$ and 20 % CO${}_{2}$). The composition of the packaging gas was determined on the basis of research result (Wang et al., 2016) and the fact that the bacteriostatic effect of CO${}_{2}$ is visible in a concentration of at least 20 % (McMillin, 2008) and increases at a reduced temperature.

### 2.2 Packaging of samples

Those intended for MAP storage were placed in trays and were packaged in polyamide/polyethylene (PA/PE) bags using the Tepro PP-5 vacuum packer. The PA/PE bags were characterized by the following gas permeability at a temperature of 23 ${}^{\circ }$C: 16–19 cm${}^{3}$/cm${}^{2}$/24 h for O${}_{2}$, 100–130 cm/cm${}^{2}$/24 h for CO${}_{2}$, 3–5 g/m${}^{2}$/4 h for N${}_{2}$ and 2–3 g/m${}^{2}$/24 h for water vapor. In the VP storage, 99 % of atmospheric air was removed from the bags, and a gas mixture composed of 80 % N${}_{2}$ and 20 % CO${}_{2}$ was injected.

The samples were stored in a chilling room with forced ventilation at 1±0.5 ${}^{\circ }$C for 10, 20 and 30 d. The quality of fresh meat, stored for approximately 48 h at 4 ${}^{\circ }$C, and VP and MAP stored meat was also compared.

### 2.3 Microbiological analyses

Microbiological assays were carried out before packaging and after opening the package MAP and VP. Samples were cut with sterile tweezers and blended with a medium at a ratio of 1 : 9. The microbiological examination consisted of inoculation with the flood method: psychrophilic and mesophilic bacteria were determined on Bullion-Agar (BA), TC (coliforms) was determined using chromo substrate, *Streptococcus faecalis* was determined using a SB medium (Slanetz-Bartley), *E. coli* was determined using an M-FC-Agar medium and *Clostridium* was determined using a Wilson–Blair medium (W.B.). Plated plates were then incubated at appropriate temperatures and incubation times (Table 1).

**Table 1:** Temperature and incubation time for determinations of individual groups of bacteria.

	Temperature	Incubation time
A 22 – psychrophilic bacteria	22 ${}^{\circ }$C	72 h
A 37 – mesophilic bacteria	37 ${}^{\circ }$C	24 h
TC – coliforms	37 ${}^{\circ }$C	48 h
FS – *Streptococcus faecalis*	37 ${}^{\circ }$C	72 h
*E. Coli*	44.5 ${}^{\circ }$C	24 h
*Clostridium*	37 ${}^{\circ }$C	24 h

The number of bacteria of individual groups was calculated based on the number of colonies grown after incubation on plates with substrates. The results were calculated using the appropriate dilutions an reported as Log10.

### 2.4 Physicochemical and sensory analyses

Natural drip loss was determined by weighing a sample of meat (about 20 g) placed in a string bag (PE) and suspended in an incubator at an air temperature of 4 ${}^{\circ }$C. After 24 h, the sample was weighed again to the nearest 0.01 g. The amount of natural drip loss (%) was calculated on the basis of the difference in weight of the sample before and after refrigerated storage.

Cooking loss was determined by cooked a weighed sample of meat (about 50 g) in plastic bags in a water bath set at 75 ${}^{\circ }$C, for 50 min. Samples were allowed to cool under cold running water for 30 min after which the cooked meat was patted dry with paper towels. The weight of each sample was recorded before and after cooking (with an accuracy of 0.01 g). Cook loss was expressed as the percentage of the weight difference.

The pH was determined by blending a 10 g sample with 100 mL deionized water for 3 min. The pH of the resultant suspension was measured after 10 min at room temperature (about 21±1 ${}^{\circ }$C) using pH meter inoLab Level 2 (WTW) and a combined electrode Polilyte Lab (Hamilton) according to PN-ISO 2917:2002 and calibrated with standard buffers of pH 4.0 and 7.0. Three readings were made for each sample, and the mean was recorded.

The meat samples were also used for shear force determination according to Honikel (1998). The cores (1.27 cm in diameter) were removed from each sample parallel to the muscle fiber axis and sheared perpendicular to this axis using INSTRON 5542 with an apparatus equipped with a Warner–Bratzler shearing device. The crosshead speed was 100 mm/min.

Color was measured using MiniScan XE Plus (Hunter Associates Laboratory Inc., Reston, VA, USA) directly on the meat surface. Results were expressed as CIE LAB (CIE, 1978): $L^{*}$ (lightness), $a^{*}$ (redness), and $b^{*}$ (yellowness). Color was evaluated using standard illumination (D65 10${}^{\circ }$ observer) with an 8 mm viewing aperture. The measurements were carried out after half an hour of holding the samples at 4 ${}^{\circ }$C covered with a membrane permeable to O${}_{2}$ and impermeable to H${}_{2}$O.

LTL were subjected to sensory evaluation to determine aroma and taste (intensity and desirability), juiciness, and tenderness of meat. Meat samples used for sensory evaluation were cooked in a 0.6 % table salt solution with a water to meat ratio of 2 : 1. After cooking, the samples were chilled to 60 ${}^{\circ }$C and subjected to taste panel evaluation by a standing committee of five evaluators in accordance with a five-point hedonic scale. The panel members were seated in individual booths in a temperature and light-controlled room and received a set of 10 samples in a completely randomized order. Sensory analysis was performed in triplicate in two sessions.

### 2.5 Statistical analyses

All statistical analyses were performed using Statistica 10.0 SoftCorp software. A two-way analysis of variance was used to examine the interaction between the type of storage and days of storage. The model used was the following:

$$y_{ijk}=\;\mu +\;S_{i}+D_{j}+S\;x\;D_{ij}+e_{ijk},$$

where $y_{ijk}$ represents each of the studied variables (e.g, pH, water loss, psychrophilic and mesophilic bacteria, $L^{*}$, $a^{*}$, $b^{*}$, and sensory evaluation), μ is least-squares mean, $S_{i}$ is the fixed effect due to the type of storage (i=1, MAP; i=2, VP), $D_{j}$ is the fixed effect due to days of storage (j=1, 0 d; j=2, 10 d; j=3, 20 d; j=4, 30 d), $S\times D_{ij}$ is the effect due to the interaction between the type of storage and days of storage, and $e_{ijk}$ is the random residual effect.

The models included all main effects and interaction terms. The significance of differences between groups was verified with Duncan's test.

## 3 Results and discussion

Changes in microbial counts are shown in Table 2. *Streptococcus faecalis, E. coli* and *Clostridium* were not found in the analyzed meat samples. The number of bacteria in the other analyzed groups was low at the beginning of the storage period, indicating a high level of hygiene during slaughter and sampling. The initial counts of psychrotrophic aerobic bacteria were $2.78{\mathrm{log}}_{10}$ CFU/g. There was no effect of the packaging method on the number of this group of bacteria. An increase (P<0.05) in the number of psychotropic aerobic bacteria with storage time from 2.78 to 3.88 was found. There was also a tendency (P=0.056) the number of bacteria in this group to increase both when MAP and VP were used. However, in the case of vacuum, higher bacterial numbers were found on storage days 20 and 30 compared to MAP. Similarly, other authors reported an increase in the number of psychotropic aerobic bacteria in VP-stored meat in comparison to MAP-stored meat (Fernández-López et al., 2008; Lorenzo and Gómez, 2012). Chilled-meat spoilage depends on the spoilage activity of psychrotrophic bacteria, which can grow in meat via undesired metabolic compounds that arise from nutrient degradation at low temperatures (Ercolini et al., 2009).

**Table 2:** Effect of vacuum packaging and modified atmosphere packaging on microbial counts (${\mathrm{log}}_{10}$ UFC/g) of fresh lamb meat during storage at 4 ${}^{\circ }$C.

Specification	Psychrotrophic	Mesophilic	Coliforms
	aerobic bacteria	aerobic bacteria	
Storage method × time			
MAP 0	2.78 ± 0.880	3.01${}^{\mathrm{ac}}$ ± 0.545	2.13${}^{\mathrm{bc}}$ ± 0.730
MAP 10	2.95 ± 0.763	2.69${}^{\mathrm{bc}}$ ± 0.489	1.80${}^{\mathrm{ab}}$ ± 0.747
MAP 20	2.96 ± 1.003	2.56${}^{\mathrm{bc}}$ ± 0.704	1.45${}^{\mathrm{ac}}$ ± 0.564
MAP 30	3.37 ± 1.020	2.46${}^{\mathrm{bc}}$ ± 0.710	1.33${}^{a}$ ± 0.465
VP 0	2.78 ± 0.880	3.01${}^{\mathrm{ac}}$ ± 0.545	2.13${}^{b}$ ± 0.730
VP 10	2.62 ± 0.528	2.30${}^{b}$ ± 0.771	1.90${}^{\mathrm{ab}}$ ± 0.585
VP 20	3.27 ± 0.968	2.84${}^{\mathrm{abc}}$ ± 0.724	2.36${}^{b}$ ± 0.797
VP 30	4.38 ± 0.763	3.33${}^{a}$ ± 0.915	3.18${}^{d}$ ± 1.189
SEM	0.101	0.075	0.093
Storage method			
MAP	3.01 ± 0.919	2.68 ± 0.636	1.68${}^{a}$ ± 0.692
VP	3.26 ± 1.042	2.87 ± 0.818	2.39${}^{b}$ ± 0.961
Time			
0	2.78${}^{a}$ ± 0.861	3.01 ± 0.533	2.13 ± 0.714
10	2.79${}^{a}$ ± 0.663	2.49 ± 0.662	1.85 ± 0.658
20	3.11${}^{a}$ ± 0.977	2.70 ± 0.713	1.91 ± 0.820
30	3.88${}^{b}$ ± 1.022	2.89 ± 0.915	2.26 ± 1.292
P values			
$P_{\mathrm{storage}\;\mathrm{method}}$	0.161	0.180	< 0.001
$P_{\mathrm{time}}$	< 0.001	0.055	0.213
$P_{\mathrm{storage}\;\mathrm{method}\;\times \;\mathrm{time}}$	0.056	0.018	< 0.001

MAP: modified atmosphere packaging (80 % N${}_{2}$ + 20 % CO${}_{2}$). VP: vacuum packaging; 0, 10, 20, 10: time of storage in days. SEM: standard error of mean. Values in each column with different letters differ significantly when P≤0.05.

The initial mesophilic aerobic bacteria counts were $3.01{\mathrm{log}}_{10}$ CFU/g (Table 2). There was no significant difference in the method of storage of meat. There was a tendency (P=0.055) in the number of bacteria of the group in relation to storage time. An interaction was found with respect to the storage method used and storage time. Mesophilic aerobic bacteria counts were significantly (P<0.05) higher in VP storage at 30 d than when using the MAP method.

The initial coliforms were $2.13{\mathrm{log}}_{10}$ CFU/g (Table 2). The MAP samples showed lower (P<0.01) counts than the VP samples. No significant differences were found between storage times. The MAP samples during the storage showed statistically highly significant lower counts of coliforms from $2.13{\mathrm{log}}_{10}$ CFU/g at day 0 to $1.33{\mathrm{log}}_{10}$ CFU/g at day 30. Counts in VP insignificantly decreased at day 10 ($1.90{\mathrm{log}}_{10}$ CFU/g) and then increased highly significantly to $3.18{\mathrm{log}}_{10}$ CFU/g at day 30. Using present MAP experimental conditions it was possible to obtain the protective effect of CO${}_{2}$ against microbial growth. According to Devlieghere et al. (1998), CO${}_{2}$ exhibits antimicrobial activity and is also partly soluble in water and fat of meat, and the solubility increases greatly with decreased temperature. Singh et al. (2011) reported that dissolved CO${}_{2}$ can increase the lag phase and generation time of microorganisms. This is confirmed by the research of Carrizos et al. (2017) on the storage of goat meat, which had a significantly lower number of aerobic mesophilic bacteria, psychrophilic bacteria and coliforms in treatments that had a higher CO${}_{2}$ concentration.

The analysis shows that both the packaging method and storage length did not significantly affect cooking loss and natural drip loss (Table 3). Other authors reported that both the packaging method and the storage time had a significant impact on cooking loss (Vergara and Gallego, 2001).

**Table 3:** Effect of vacuum packaging and modified atmosphere packaging on physicochemical parameters of lamb meat during storage at 4 ${}^{\circ }$C.

Specification	Cooking loss	Natural drip	pH	Shear force
	(%)	loss (%)		(N)
Storage method × time				
MAP 0	38.87 ± 2.738	1.15 ± 0.443	5.68 ± 0.084	21.70 ± 5.355
MAP 10	39.89 ± 1.902	1.15 ± 0.432	5.73 ± 0.134	20.41 ± 6.755
MAP 20	39.93 ± 1.965	1.30 ± 0.196	5.71 ± 0.063	20.66 ± 5.017
MAP 30	40.39 ± 2.528	1.12 ± 0.443	5.72 ± 0.062	18.00 ± 2.866
VP 0	38.87 ± 2.738	1.15 ± 0.443	5.68 ± 0.084	21.70 ± 5.355
VP 10	39.32 ± 3.481	1.19 ± 0.481	5.87 ± 0.106	20.24 ± 7.658
VP 20	40.60 ± 2.843	1.06 ± 0.263	5.83 ± 0.151	17.84 ± 3.707
VP 30	40.68 ± 1.944	1.41 ± 0.457	5.89 ± 0.159	14.76 ± 3.446
SEM	0.262	0.041	0.014	0.093
Storage method				
MAP	39.77 ± 2.306	1.18 ± 0.477	5.71${}^{a}$ ± 0.090	20.19 ± 5.203
VP	39.87 ± 2.829	1.20 ± 0.427	5.82${}^{b}$ ± 0.149	18.63 ± 5.787
Time				
0	38.87 ± 2.678	1.15 ± 0.433	5.68${}^{a}$ ± 0.082	21.70${}^{b}$ ± 5.237
10	39.60 ± 2.759	1.17 ± 0.448	5.80${}^{b}$ ± 0.138	20.32${}^{b}$ ± 7.062
20	40.26 ± 2.414	1.18 ± 0.256	5.77${}^{b}$ ± 0.130	19.25${}^{\mathrm{ab}}$ ± 4.549
30	40.53 ± 2.210	1.27 ± 0.465	5.80${}^{b}$ ± 0.145	16.38${}^{a}$ ± 3.514
P values				
$P_{\mathrm{storage}\;\mathrm{method}}$	0.854	0.754	< 0.001	0.149
$P_{\mathrm{time}}$	0.119	0.755	0.001	0.006
$P_{\mathrm{storage}\;\mathrm{method}\;\times \;\mathrm{time}}$	0.863	0.179	0.056	0.592

MAP: modified atmosphere packaging (80 % N${}_{2}$ + 20 % CO${}_{2}$). VP: vacuum packaging. 0, 10, 20, 10: time of storage in days. SEM: standard error of mean. Values in each column with different letters differ significantly when P≤0.05.

The initial pH value was 5.68, very similar to those reported by other authors in lamb meat (Bórnez et al., 2010; Gutiérrez et al., 2011; Wang et al., 2016). The obtained pH values ranged from 5.68 to 5.88 and insignificantly exceeded the normal pH values for lamb (5.5–5.8) given by Silva Sobrinho et al. (2005). The pH values showed highly significant differences (P<0.01) depending on both storage time and package treatment. The mean pH value of MAP meat was 5.71, while VP meat was pH 5.81. The fact that the MAP meat pH value was significantly lower might be related to the presence of CO${}_{2}$ in this package type. Leygonie et al. (2011) postulated that the CO${}_{2}$ dissolves into meat fat and water phases, associates with H${}^{+}$ and forms carbonic acid, which causes a pH decrease. However, pH value can be affected by many other factors. Many other authors also noticed higher pH values at the end of storage in samples from vacuum-packed meat (Lorenzo and Gómez, 2012), although other studies showed no differences between VP and MAP lamb meat (Wang et al., 2016). In the present study, pH increased during the storage time from 5.68 to 5.80, unlike in other studies where pH value decreased during storage (Wang et al., 2016). However, other authors showed no differences in pH value stemming from the packaging method or storage time (Bórnez et al., 2016). In similar studies on the effect of vacuum and modified atmosphere packaging methods on fermented ham, Jin and Choi (2017) also observed a statistically significant impact of both storage time and packaging method. A tendency was noted (P=0.056) regarding the interaction of the packaging method used with storage time. The VP samples showed higher pH values at 10, 20 and 30 d of storage in comparison to the ones from MAP during the storage.

Warner–Bratzler shear force values did not differ (P=0.149) depending on the type of packaging method. Depending on the storage time, a lower (P<0.01) value of this trait was found on day 30. The initial shear force value was 21.70 N and decreased after 30 d of storage in MAP treatment to 18.00 N and after 30 d of storage in VP treatment to 14.76 N. However, the interaction was not found (P=0.592). Other authors also found no significant differences among packaging types and during storage time relating to Warner–Bratzler shear force values (Vergara and Galleo, 2001).

**Table 4:** Effect of vacuum packaging and modified atmosphere packaging on the meat color of lamb meat during storage at 4 ${}^{\circ }$C.

Specification	$L^{*}$	$a^{*}$	$b^{*}$
Storage method × time			
MAP 0	46.29 ± 1.818	11.92 ± 0.984	14.47 ± 0.803
MAP 10	45.38 ± 2.121	13.10 ± 1.169	14.61 ± 1.014
MAP 20	45.51 ± 3.076	13.02 ± 1.197	14.60 ± 1.281
MAP 30	45.10 ± 1.818	13.13 ± 0.904	14.79 ± 0.741
VP 0	46.29 ± 1.818	11.92 ± 0.984	14.47 ± 0.803
VP 10	46.14 ± 1.678	12.55 ± 0.677	14.28 ± 1.001
VP 20	45.80 ± 2.528	13.39 ± 1.044	15.03 ± 1.149
VP 30	45.63 ± 2.464	13.52 ± 0.884	15.14 ± 0.660
SEM	0.221	0.114	0.098
Storage method			
MAP	45.57 ± 2.239	12.79 ± 1.154	14.62 ± 0.958
VP	45.96 ± 2.103	12.85 ± 1.096	14.73 ± 0.966
Time			
0	46.29 ± 1.778	11.92${}^{a}$ ± 0.963	14.47 ± 0.786
10	45.76 ± 1.910	12.83${}^{b}$ ± 0.975	14.44 ± 1.000
20	45.65 ± 2.757	13.20${}^{b}$ ± 1.115	14.81 ± 1.211
30	45.36 ± 2.134	13.33${}^{b}$ ± 0.896	14.97 ± 0.709
P values			
$P_{\mathrm{storage}\;\mathrm{method}}$	0.393	0.795	0.560
$P_{\mathrm{time}}$	0.529	< 0.001	0.160
$P_{\mathrm{storage}\;\mathrm{method}\;\times \;\mathrm{time}}$	0.943	0.326	0.488

MAP: modified atmosphere packaging (80 % N${}_{2}$ + 20 % CO${}_{2}$). VP: vacuum packaging. 0, 10, 20, 10: time of storage in days. SEM: standard error of mean. Values in each column with different letters differ significantly when P≤0.05.

According to McMillin (2008), meat purchasing decisions are more influenced by color than any other quality factor, and thus color stability or discoloration is the most important quality attribute for shelf life. The effect of packaging methods on the color of lamb meat is shown in Table 4. No interactions were observed between the treatments and storage time (P>0.05). Lightness ($L^{*}$), redness ($a^{*}$) and yellowness ($b^{*}$) did not show significant differences (P>0.05) due to packaging conditions or due to storage time. Myoglobin oxygenation is visible to about 5 mm deep within the meat. Color occurring on the surface depends on the chemical state of this pigment and the balance in the availability of O${}_{2}$ and tissue respiration (Jose et al., 2009). Maintaining a stable meat color using vacuum packaging and in a modified atmosphere (80 % N + 20 % CO${}_{2}$) may be the result of the unavailability of oxygen. The presence of high level of O${}_{2}$ in MAP gas mixtures increases lipid oxidation in meat and meat products which can cause rancidity increase and may enhance myoglobin oxidation (Faustman et al., 2010; Lund et al., 2007). Oxymyoglobin to metmyoglobin conversion reduces redness and leads to meat color deterioration (Insausti et al., 1999). In the present experiment, the use of vacuum packaging and a modified atmosphere of gases (without oxygen) provided an adequate anaerobic environment to prevent pigment oxidation. Only an increase in red saturation (P<0.001) under the influence of storage time was observed, the meat was initially characterized by less saturation with this pigment (11.92) compared to meat after 10 (12.83), 20 (13.20) and 30 (13.33) d of storage. Obtained results of redness ($a^{*}$) are in line with results of Morales-delaNuez et al. (2009) on the effect of vacuum or modified atmosphere packaging on goat meat. However, on the other hand, Gutiérrez et al. (2011) observed decreases in redness during the storage of Merino lamb meat. The bright red color of meat is considered to be a positive aspect because it is associated with freshness and the highest-quality product (Berruga et al., 2005). The proportion of red color in meat depends on many factors, including the transformation of myoglobin, oxymyoglobin, metmyoglobin and carboxymyoglobin (McMillin, 2008). In addition, Vergara and Gallego (2001) did not report changes in $L^{*}$ and $b^{*}$ values during storage in pork meat packed in modified atmosphere (40 % CO${}_{2}$ + 60 % N${}_{2}$) similar to the present experiment (20 % CO${}_{2}$ + 80 % N${}_{2}$). Hur et al. (2013) also did not observe the impact of storage time and vacuum and modified atmosphere (30 % CO${}_{2}$ + 70 % N${}_{2}$) on CIE LAB parameters of low-grade beef. Other authors who performed research on the refrigerated storage of lamb meat in a vacuum and in a modified gas atmosphere have shown an effect on changes in $L^{*}$, $a^{*}$, $b^{*}$ parameters in the CIE LAB system both due to the influence of storage time and depending on the packaging method used (Berruga et al., 2005; Karabagias et al., 2011; Soldatou et al., 2009; Wang et al., 2016).

**Table 5:** Effect of vacuum packaging and modified atmosphere packaging on the sensory properties of lamb meat during storage at 4 ${}^{\circ }$C.

Specification	Aroma	Aroma	Juiciness	Tenderness	Taste	Taste
	intensity	desirability			intensity	desirability
Storage method × time						
MAP 0	4.58 ± 0.289	4.54 ± 0.334	3.92 ± 0.417	3.88 ± 0.483	4.21 ± 0.396	4.25 ± 0.399
MAP 10	4.58 ± 0.359	4.67 ± 0.246	4.04 ± 0.722	4.17 ± 0.651	4.17 ± 0.444	4.08 ± 0.359
MAP 20	4.54 ± 0.450	4.46 ± 0.450	3.58 ± 0.417	3.71 ± 0.450	4.00 ± 0.477	3.83 ± 0.537
MAP 30	4.38 ± 0.377	4.38 ± 0.377	3.75 ± 0.337	3.75 ± 0.261	4.13 ± 0.377	3.92 ± 0.469
VP 0	4.58 ± 0.289	4.54 ± 0.334	3.92 ± 0.417	3.88 ± 0.483	4.21 ± 0.396	4.25 ± 0.399
VP 10	4.71 ± 0.334	4.71 ± 0.334	4.58 ± 0.469	4.63 ± 0.433	4.46 ± 0.396	4.46 ± 0.396
VP 20	4.46 ± 0.334	4.42 ± 0.359	3.96 ± 0.498	4.00 ± 0.477	4.04 ± 0.334	4.00 ± 0.369
VP 30	4.46 ± 0.334	4.42 ± 0.359	4.25 ± 0.399	4.46 ± 0.334	4.29 ± 0.257	4.25 ± 0.261
SEM	0.036	0.036	0.055	0.055	0.041	0.045
Storage method						
MAP	4.52 ± 0.357	4.51 ± 0.365	3.82${}^{b}$ ± 0.510	3.88${}^{b}$ ± 0.500	4.13 ± 0.461	4.02${}^{b}$ ± 0.461
VP	4.55 ± 0.330	4.52 ± 0.357	4.18${}^{a}$ ± 0.510	4.24${}^{a}$ ± 0.526	4.25 ± 0.371	4.24${}^{a}$ ± 0.386
Time						
0	4.58 ± 0.282	4.54${}^{\mathrm{ab}}$ ± 0.327	3.92${}^{b}$ ± 0.408	3.88${}^{b}$ ± 0.472	4.21 ± 0.388	4.25${}^{a}$ ± 0.390
10	4.65 ± 0.345	4.69${}^{a}$ ± 0.288	4.31${}^{a}$ ± 0.656	4.40${}^{a}$ ± 0.589	4.31 ± 0.434	4.27${}^{a}$ ± 0.416
20	4.50 ± 0.361	4.44${}^{b}$ ± 0.399	3.77${}^{b}$ ± 0.489	3.85${}^{b}$ ± 0.477	4.02 ± 0.466	3.92${}^{b}$ ± 0.458
30	4.42 ± 0.351	4.40${}^{b}$ ± 0.361	4.00${}^{b}$ ± 0.442	4.10${}^{b}$ ± 0.466	4.21 ± 0.369	4.08${}^{\mathrm{ab}}$ ± 0.408
P values						
$P_{\mathrm{storage}\;\mathrm{method}}$	0.662	0.885	0.001	< 0.001	0.120	0.001
$P_{\mathrm{time}}$	0.126	0.026	0.001	< 0.001	0.080	0.010
$P_{\mathrm{storage}\;\mathrm{method}\;\times \;\mathrm{time}}$	0.739	0.973	0.187	0.064	0.563	0.366

MAP: modified atmosphere packaging (80 % N${}_{2}$ + 20 % CO${}_{2}$). VP: vacuum packaging. 0, 10, 20, 10: time of storage in days. SEM: standard error of mean. Values in each column with different letters differ significantly when P≤0.05.

An analysis of the sensory properties of meat (Table 5) showed no significant differences regarding the interaction of the packaging method used with storage time. The packaging method had an influence (P<0.05) on juiciness, tenderness and taste desirability. Vacuum-packed meat was characterized by higher scores in comparison to MAP meat. In addition, storage time had a significant impact on some sensory characteristics (P<0.05). The meat was rated highest after 10 d of storage for juiciness, tenderness, and aroma and taste desirability. Berruga et al. (2005), who analyzed both the effects of vacuum packing and modified gas atmosphere on lamb meat, reported that vacuum-packed meat maintained desirable sensory characteristics better than meat packed under a modified atmospheres.

## 4 Conclusions

In conclusion, vacuum and modified atmosphere packaging extend storage time without damaging the quality of lamb meat; however, further MAP studies are needed to develop adequate methods for the packaging of lamb meat.

An analysis of the physicochemical parameters, sensory properties and color of lamb meat showed no significant differences regarding the interaction between the packaging method used and storage time. There was an interaction between storage time and packaging method regarding mesophilic aerobic bacteria and coliform counts. Both storage time and packing method significantly affected pH, juiciness, tenderness and the desirability of the meat's taste. However, it was found that storage time significantly affected the number aerobic psychrotrophic bacteria, shear force value, redness, and the desirability of the meat's aroma and taste. The packaging method only further affects the quantity of coliform counts.

## Data Availability

The data are available upon reasonable request from the corresponding author.
